# Popular Pastimes as Aids to Hospital Funds

**Published:** 1889-11-23

**Authors:** A. W. Webster

**Affiliations:** late Chairman of Workmen's Governors Sunderland Infirmary, now Assistant Secretary London Hospital Saturday Fund


### Popular Pastimes as Aids to Hospital Funds.

By A. W. Webster, late Chairman of Workmen's Governors Sunderland Infirmary, now Assistant Secretary London Hospital Saturday Fund.

Swift and far-reaching are the changes which science, literature, and commerce have undergone in the past few years, and he who would travel with the speed and spirit of his age must ruthlessly tear himself from the stage-coach pace of his forefathers and adopt something approaching that of the "Flying Scotsman" that ploughs the air at a mile a minute.

In the midst of this whirlwind of excitement, many may be inclined to pause at the heading of these articles and exclaim "Pastime, forsooth, no such luxury exists in our day and generation!" To calm the righteous indignation of our modern go-ahead Englishman, a definition of my title may be necessary. By pastimes I mean not only those games and exercises prescribed by physiologists as essential to health and longevity, but also those changes of occupation so necessary to relieve the monotony of daily life, and so often indulged in by our busiest and wisest men and women.

It will be obvious to all that this appeal is not only made to the cricketer, the football player, the cyclist, the musician, the elocutionist, and those actually engaged in the various pastimes hereafter enumerated, but is also meant to reach the patrons and supporters who so generously and often enthusiastically supply the monetary aids to such movements. Galas, concerts, bazaars, etc., in aid of different charities are already so common, that I need make no apology for attempting to gather together the few simple schemes coming under my own observation, and submitting them to my readers in a practical form. It is possible, however, that some of our more sensitive friends may be inclined to cry out against the very idea of seeking to augment the funds of so sacred an institution from the pleasures and pastimes of the people. I may confess at the outset that whatever prejudice I formerly had on this point has gradually vanished as experience increased. Having concluded that any properly-conducted extraneous effort on behalf of a beneficent institution usually generates good feeling, secures increased interest and support, ennobles and sanctifies the particular pastime indulged in, and nearly always results in benefiting suffering humanity, I have little hesitation in proceeding with a self-imposed task, which I imagine the exigencies of present-day hospital finance demand.

Everyone knows that our purely voluntary system of maintaining charitable institutions has long been the pride and admiration of the world. Will this admired system of ours stand the test of time? Are there not already indications of change? To test the question, let me ask how many unendowed charities can be found entirely kept up by unsolicited spontaneous offerings?

My previous articles have dealt so fully with methods of raising incomes direct from the working classes, that I do not propose to revert to that topic at present, but will rather offer suggestions as to how direct subscriptions may be supplemented by indirect ones from the same class of people.

It must not be understood from this that I consider obligation is confined to patients alone, or even to the class from whom they are drawn. Modern hospitals are so much interwoven with our social system, that no true-hearted Briton will presume to hint at how we could get along without them. Hospitals cannot be dispensed with, and the sooner the general public awaken to this fact the better. If it be impossible to support them by voluntary contributions, then they must be kept up by compulsory rates.

As many of the games and pastimes I propose to refer to will appeal to the "masses" rather than the "classes" for support, it will not be out of place for me to reiterate the desirability, nay, the necessity, of giving workmen a share in all hospital management. Even when this point is universally granted, only a few of the workmen's representatives can ever be placed on the executive committees of the various institutions, and it is one of the objects of these articles to suggest supplementary movements in which the remaining delegates, as well as others interested in the work, may constantly find spheres of activity and usefulness.

The officials of any club, society, or organisation, who propose initiating charity games or performances on their own responsibility will do well to attend to these preliminaries. They will find the workmen's representatives of immense value in circulating information, selling tickets of admission, and promoting the interest and success of all their undertakings.

### Cricket.

There are few Englishmen, possessed of ordinary physical fitness, who have not at some period of life indulged in the national game of cricket. I presume there are no towns, and very few villages in the country, without their recognised cricket clubs, the more popular and influential of which are usually conducted under local patronage. As thorough organisation and good management is the rule rather than the exception with these, it ought to be a comparatively easy task to secure wide interest and a large attendance of spectators to witness a good match.

To all lovers of the game I would offer a single suggestion, and would ask the favour of an honest experiment. It is neither novel nor elaborate, and consists of a simple request that negotiations be opened with the various club officials with a view to getting them to arrange a few really good matches on behalf of local charities each season. Nothing will tend to popularise the game more speedily. The shyest member of the club interested, the least effective bowler, the most awkward batsman will vie with local "Graces" in issuing invitations broad-cast to attend a match in aid of a good object, when nothing else would induce them to press the claims of their club on the public.

The most popular clubs known to the writer follow the above plan, and regularly place two or three first-class charity matches on their fixture cards for each season. I am much mistaken if a simple request to the more prominent officials connected with the chief clubs in any town be not sufficient to establish this desirable practice universally, and which is sure to result in considerable aid being given to the maintenance of local charities.

In a certain northern town it is the practice of the portly aldermen to face the agile councillors with bat and ball at least once a year, in aid of a local charity. The day is one of rejoicing to the stately functionaries and their numerous friends. Conventionalities are laid aside, mirth and jocularity everywhere abound. High-class cricket is unavoidably sacrificed for an equally popular class, which we may designate "queer." The finer points of the game, such as "no balls" and "wides," are beyond the knowledge or beneath the notice of the extemporised cricketers. The ludicrous attitude of a fat alderman making desperate attempts at thrashing the leather occasionally creates a roar of laughter, and when a lucky sky-hit enables a popular councillor to score a "single," it is looked upon as a feat of no mean order. The net result of the game is usually stiff joints and tired muscles to the municipal performers, immense pleasure to the public, and a ten or twenty pound note handed over to some worthy charity. The dignity of the local rulers does not seem to suffer in the ordeal, and I see no reason why the practice should not spread to other places.

In the same town, the practitioners of law and medicine annually enjoy an afternoon's amusement of a like description. Although the game sometimes provides a little professional practice for the doctors, it has never been known to supply briefs to the barristers.

With a little judicious management, it is often possible to arrange a friendly game, in aid of a charity, between two theatrical or concert companies who may happen to be staying for the time in the same town. I dare not hint that actors are expert cricketers, but their facility of resource will usually make them equal to creating an interesting game. The artistic somersault of the clown, the revolving cart-wheel action of the harlequin, the inimitable tricks of the juggler, the stately bearing of the comedian interwoven with an ordinary game of cricket, will always prove an irresistible attraction to certain portions of the community who can generally find leisure and spare sixpences for such exhibitions. Drapers and grocers, butchers and bakers, soldiers and volunteers, and a host of others can be brought together in this manner, each being able to influence an attendance of their own particular friends and acquaintances, as well as a goodly number of the general public.

(*To be continued.*)

